# The Association between Intelligence Scores and Family History of Psychiatric Disorder in Schizophrenia Patients, Their Siblings and Healthy Controls

**DOI:** 10.1371/journal.pone.0077215

**Published:** 2013-10-09

**Authors:** Kim H. W. Verweij, Eske M. Derks, Genetic Risk and Outcome in Psychosis (GROUP) investigators

**Affiliations:** 1 Department of Psychiatry, Rudolf Magnus Institute of Neuroscience, University Medical Centre Utrecht, Utrecht, the Netherlands; 2 Department of Psychiatry, Academic Medical Center, University of Amsterdam, Amsterdam, The Netherlands; Peking University, China

## Abstract

**Background:**

The degree of intellectual impairment in schizophrenia patients and their relatives has been suggested to be associated with the degree of familial loading for schizophrenia. Since other psychiatric disorders are also more present in relatives of schizophrenia patients, the definition of family history should be broadened. The association between family history for psychiatric disorder and intelligence scores was investigated in patients with non-affective psychosis, their unaffected siblings and controls.

**Methods:**

A sample of 712 schizophrenia proband families (696 patients and 766 siblings) and 427 healthy control families (517 subjects) participated in this study. Family history of psychiatric disorder was determined while excluding the data of the participating schizophrenia patient. A dichotomous division was made between families with no first- or second degree relative with psychiatric disorder and families with one or more affected relatives. Total intelligence scores were estimated by admission of the short form of the Wechsler Adult Intelligence Scale III.

**Results:**

A significant interaction was found between family history of psychiatric disorder and clinical status (F(2,1086.87)= 4.17; p=.016). Patients with a positive family history of psychiatric disorder obtained higher intelligence scores compared to patients with no family history (mean IQ scores are 95.52 and 92.72) with an opposite effect in controls (mean IQ scores are 108.71 and 111.19). No significant difference was found between siblings of schizophrenia patients with or without a positive family history (mean IQ scores are 102.98 and 103.24).

**Conclusion:**

In patients with schizophrenia, a negative family history of psychiatric disorder was associated with relatively low IQ suggesting that the etiology in these patients may involve environmental or genetic factors which are unique to the patient and are not observed in other relatives. Possible factors include severe environmental stressors containing premature birth or brain injury and genetic factors (e.g de novo Copy Number Variants).

## Introduction

Schizophrenia is characterized by general intellectual deficits. A consistent finding is an impaired IQ score in patients compared to controls [1–3]. These lower IQ scores can be present prior to the manifestation of the illness [4] and persist in most patients despite improvement of symptoms. Poor cognitive performance has also been found in siblings as compared to healthy control subjects [5,6] and in offspring of patients with schizophrenia [7–9]. These findings suggest that intellectual impairment in schizophrenia has a familial component and can be seen as a genetically mediated risk indicator for schizophrenia [10]. Moreover, lower IQ scores in patients are also seen in other psychiatric disorders such as affective, personality and anxiety disorders [11].

It has been hypothesized that the degree of intellectual impairment is associated with the degree of genetic loading [12]. Since there is no golden standard measure for genetic loading, family history data for psychosis or schizophrenia are frequently used. A group of special interest are those families densely affected by psychosis. Maziade and colleagues demonstrated that patients and unaffected relatives from such families have lower global IQ scores compared to healthy controls [13]. Remarkably, these findings are not exclusive for patients and relatives from densely affected families. Schizophrenia patients with only one affected relative also have impaired IQ scores compared to healthy controls [14] as well as unaffected subjects with one first- or second-degree relative with schizophrenia [15–17]. Only a few studies (n=56-154 subjects) have compared IQ scores between schizophrenia patients with and without relatives affected by psychosis [18–20]. Interestingly, most of these studies did not detect a difference between full scale IQ scores for patients with or without affected relatives in the family [19,20]. This is consistent with the conclusion that there are few robust neurocognitive differences between schizophrenia patients with and without relatives affected by psychosis [21,22]. However, Norman and colleagues found higher IQ scores in patients without affected relatives [18].

Although previous studies have assessed family history based on the number of individuals with psychosis, recent findings indicate that the risk of clinically diagnosed schizophrenia is associated with a family history of a much wider range of psychiatric disorders [23]. Moreover, a variety of other psychiatric disorders are often present in relatives of patients with schizophrenia [24]. High rates of axis I disorders such as attention deficit hyperactivity disorder, mood disorder and anxiety disorder have been shown in offspring and siblings from patients with schizophrenia [25–27]. Also, major depressive disorder and substance abuse are frequently reported in first degree relatives of first episode psychotic patients [24] and finally, relatives have an increased prevalence of schizotypal, paranoid, schizoid and avoidant personality disorder [28]. This implies an increased vulnerability for a wide variety of psychiatric disorders in families of schizophrenia patients. The increased vulnerability for various psychiatric disorders is further supported by recent findings indicating that the genetic risk factors for schizophrenia are partly shared with the genetic vulnerability for other psychiatric disorders including bipolar disorder [29], depression [30], schizoaffective and manic disorder [31]. Therefore, it could be hypothesized that the definition of familial loading based on the presence or absence of psychosis in families of patients with schizophrenia should be broadened by focusing on psychiatric disorder in general irrespective of a specific diagnosis.

Since impaired IQ scores are found in different psychiatric disorders and various psychiatric disorders are often present in relatives of patients with schizophrenia, the aim of this study is to investigate the association between family history for psychiatric disorder and IQ in schizophrenia patients, their non-psychotic siblings and healthy controls. Family history of psychiatric disorder was determined based on the presence or absence of psychiatric disorder in the first- and second-degree relatives of 717 schizophrenia patient families and 427 healthy control families.

## Methods

### Subjects

The data pertain to baseline measures of an ongoing longitudinal multicenter study Genetic Risk and Outcome in Psychosis (GROUP) [32]. The sample was recruited in the Netherlands and Belgium. Patients with non-affective psychosis were identified through clinicians working in regional mental health services whose caseloads were screened for inclusion criteria. Subsequently, a group of patients presenting consecutively at these services as either outpatients or inpatients were recruited for the study. Family members were recruited through participating patients. Healthy controls were selected through a system of random mailings to addresses in the catchment areas of the patients. Inclusion criteria were (1) age range of 16 to 50 years; (2) good command of the Dutch language; and (3) being able and willing to give informed consent. Patients had to meet the DSM-IV criteria for non-affective psychotic disorder, as assed by clinical interview with the Comprehensive Assessment of Symptoms and History (CASH) [33]. The unaffected sibling and healthy control status was defined by the absence of any lifetime psychotic disorder by CASH interview. An additional inclusion criterion for the control group was no first or second degree relatives with psychotic disorder.

At baseline, the total GROUP sample consisted of 1120 patients with non-affective psychotic disorder, 1057 of their siblings and 590 unrelated controls. For the present study we excluded data from participants with incomplete information on family history of psychiatric disorder. This resulted in the exclusion of 239 patients, 153 siblings and 62 controls. In addition, IQ scores were missing or incomplete for 46 patients, 15 siblings and 6 controls and these subjects, therefore, were also excluded from this study. Patients (n=139) and their siblings (n=123) with missing DSM IV diagnosis or a diagnosis of delusional disorder or psychotic disorder in the context of substance abuse or somatic disorder were excluded because the relationship with impaired cognitive functioning and increased vulnerability for psychiatric disorder is unclear. Finally, there were 5 healthy controls with a first- or second-degree relative with psychosis: they were excluded as they did not meet inclusion criteria. In total 696 patients, 766 siblings from 712 families and 517 healthy controls from 427 families participated in the study. The patients had a mean age of 27.0 years (SD =7.1) and 78% was male. Siblings had a mean age of 27.5 years (SD =8.1) and 46% was male. Controls had a mean age of 30.4 years (SD =10.6) and 46% was male. The majority of the patients (81%) were diagnosed with schizophrenia, while the remaining patients were diagnosed with schizophreniform disorder (6%), brief psychotic disorder (2%) or psychotic disorder not otherwise specified (11%). The majority of the siblings (86.8%) and controls (90.7%) had no lifetime psychiatric diagnosis. The remaining siblings were diagnosed with depression (11.5%), bipolar disorder (0.7%), autism (0.4%), adjustment disorder (0.3%), anorexia nervosa (0.3%) and personality disorder (0.1%). The remaining controls were diagnosed with depression (8.7%), bipolar disorder (0.2%), adjustment disorder (0.2%) and obsessive compulsive disorder (0.2%).

### Ethics statement

The study was approved by the standing ethics committee “Medisch Ethische Toetsingscommissie (METC) Utrecht”. All of the subjects gave written informed consent in accordance with the committee’s guidelines. When participants aged 16 or 17 were included, written informed consent was also given by a parent or caretaker. Patients with non-affective psychosis were identified through clinicians working in regional mental health services. These clinicians assessed if patients were mentally competent and asked if they were interested in the study. When they were interested, the study was verbally explained to them by a research employee and the informed consent was given to the patient to read at home. Only when the patient was willing to participate in the study, the family members were approached. Participation in this study was not interfering with treatment. All potential participants who declined to participate or otherwise did not participate were eligible for treatment (if applicable) and were not disadvantaged in any other way by not participating in the study.

### Family history of psychiatric disorder

The Family Interview for Genetic Studies (FIGS) was administered [34] to assess the presence of psychiatric disorders including psychosis, depression, mania and alcohol or drug abuse. In patient families, the first informant of choice for the FIGS interview was the mother. When the mother was not willing or able to provide family history information, the father or sibling of the patient was interviewed. In control families, family history information was usually provided by the participating control. As this may cause systematic differences between patient and control families, a subset (N=33) of the mothers from the healthy controls was approached and interviewed using the FIGS. Family history of psychiatric disorder was determined while excluding the data of the participating schizophrenia patient. In patients, relatives as well as in controls a dichotomous division was made between individuals with no first or second degree relative with psychiatric disorder (excluding the proband schizophrenia patient) and individuals with one or more affected relatives. A within and not a between group comparison was made. That is, in patients a comparison is made between patients with and without affected relatives. In siblings, siblings with a single affected relative were compared to siblings with multiple affected relatives. The same comparison was made for controls. No comparisons were made between patients on the one hand and siblings or controls on the other hand. Since controls were selected for absence of psychosis in first and second degree relatives, the positive family history group in controls comprises family members affected with depression, mania and alcohol or drug abuse.

### IQ

Total IQ was estimated by admission of the short form of the Wechsler Adult Intelligence Scale III (WAIS III) [35]. All participants completed the subscales Arithmetic, Digit Symbol-Coding, Block Design and Information [36]. Following the example of Blyler (2000), IQ was calculated by the weighted average of the scaled scores multiplied by 11/4 as the WAIS comprises 11 subscales to calculate total IQ scores while only 4 of them are completed by the participant.

### Analysis

Data were analyzed using SPSS version 20.0 for Windows. To investigate differences in demographic variables, ANOVA was used for continuous variables and χ^2^ test was used for categorical variables. The main outcome measure in this study is the IQ score of patients, siblings, and controls with and without a family history of psychiatric disorder. Linear Mixed model analysis was used to investigate differences in IQ scores including clinical status, family history for psychiatric disorder (present/absent), and the interaction effect between clinical status and family history as independent variables while controlling for dependency of the data due to familial relatedness. As a covariance type, compound symmetry was used. Gender and psychiatric diagnosis of the subject were included as covariates in this analysis. A type-I error rate of 0.05 was used.

## Results

### Sample

Patients and siblings with a family history of psychiatric disorder were significantly younger compared to patients (F(1,695)=7.72; p=.01) and siblings (F(1,765)=4.40, p=.04) with no family history. However, the differences are small. The mean age in patients with and without a family history is 26.5 years [25.9-27.1] and 28.2 years [27.1-29.2], respectively, while the respective mean ages of siblings are 27.1 years [26.4-27.8] and 28.4 years [27.4-29.4]. In controls, age was not significantly different for participants with and without a family history of psychiatric disorder. We investigated whether age of onset differences explained the significant difference in age between patients with and without affected relatives. Age of onset was not significantly different between patients with and without relatives affected by psychiatric disorder and we therefore did not include age of onset as a covariate in statistical analyses. In siblings with a family history of psychiatric disorder the highest degree of education was lower and the prevalence of a lifetime diagnosis for a psychiatric disorder was significantly higher compared to siblings with a negative family history of psychiatric disorder. No such differences were found between patients or controls with and without a family history of psychiatric disorder. Gender, parental education and gross income a month were not significantly different between family history groups. The proportion of patient families assigned to the group with a family history of psychiatric disorder (70%) was higher than the proportion of control families (43%) which is expected as schizophrenia is associated with an increased prevalence of psychopathology in the relatives of patients. Sample characteristics of the patient and control families and the participating patients, siblings and controls in the positive and negative family history groups are summarized in tables 1 and 2.

**Table 1:** Descriptives of the negative (FH-) and positive (FH+) family history groups.

	**FH- patient families**	**FH+ patient families**	**FH- control families**	**FH+ control families**
**Number of families**	214	498	244	183
**Mean amount of family members**	17.84 (SD =7.44)	18.31 (SD =7.10)	18.46 (SD =8.76)	18.26 (SD =7.32)
**Relatives with psychosis**	n.a.	N=219 (2.40%)	n.a.	n.a.
**Relatives with depression**	n.a.	N=789 (8.65%)	n.a.	N=258 (7.72%)
**Relatives with mania**	n.a.	N=68 (0.75%)	n.a.	N=14 (0.42%)
**Relatives with substance abuse**	n.a.	N=208 (2.28%)	n.a.	N=64 (1.92%)

**Table 2:** Descriptives of the participants in the negative (FH-) and positive (FH+) family history group.

				**Statistics**		
		**FH -**	**FH +**	***F (df=1)***	**X^2^(*df=1*)**	***p***
**Patients**	**N**	207	489			
	**Age**	28.17 (SD =7.78)	26.54 (SD =6.77)	7.72		.01
	**Gender, male**	78.3%	77.9%		.01	.92
	**Education, highest degree^a^**	4.20 (SD =2.09)	3.94 (SD =2.03)		2.47	.12
	**Parental education^a^**	4.64 (SD =2.18)	4.52 (SD =2.20)		.34	.56
	**Gross income a month^b^**	1.14 (SD =.79)	1.08 (SD =.78)		0.58	.45
	**Mean IQ score**	92 (SD =15.7)	95 (SD =16.1)	4.47		.04
	**Diagnosis, % schizophrenia**	N=172 (83.1%)	N=394 (80.6%)		.61	.44
	**Age of onset first psychosis**	22.9 (SD =7.1)	21.9 (SD =6.4)	3.22		.07
**Siblings**	**N**	241	525			
	**Age**	28.40 (SD =7.82)	27.09 (SD =8.07)	4.40		.04
	**Gender, male**	49.0%	45.1%		.97	.33
	**Education, highest degree^a^**	5.31 (SD =2.02)	4.98 (SD =2.13)		3.81	.05
	**Parental education^a^**	4.64 (SD =2.19)	4.58 (SD =2.25)		.10	.75
	**Gross income a month^b^**	1.93 (SD =.87)	1.80 (SD =1.01)		1.97	.16
	**Mean IQ score**	103 (SD =16.1)	102 (SD =15.4)	.00		.95
	**Lifetime diagnosis, % yes**	N=19 (7.9%)	N=82 (15.6%)		8.6	.00
	**No diagnosis**	N=222 (92.1%)	N=443 (84.4%)			
	**Bipolar**	n.a.	N=5 (1.0%)			
	**Depression**	N=18 (7.5%)	N=70 (13.3%)			
	**Other^c^**	N=1 (0.4%)	N=7 (1.3%)			
**Controls**	**N**	304	213			
	**Age**	30.38 (SD =11.10)	30.45 (SD =9.85)	.01		.94
	**Gender, male**	45.1%	46.0%		.05	.83
	**Education, highest degree^a^**	5.30 (SD =1.75)	5.59 (SD =1.75)		3.44	.06
	**Parental education^a^**	4.20 (SD =2.17)	4.42 (SD =2.22)		1.25	.26
	**Gross income a month^b^**	1.85 (SD =.86)	1.68 (SD =1.04)		2.82	.09
	**Mean IQ score**	111 (SD =14.8)	108 (SD =15.1)	3.47		.06
	**Lifetime diagnoses, % yes**	N=30 (9.7%)	N=18 (8.5%)		.30	.59
	**No diagnosis**	N=274 (90.1%)	N=195 (91.5%)			
	**Bipolar**	n.a.	N=1 (0.5%)			
	**Depression**	N=30 (9.9%)	N=15 (7.0%)			
	**Other^c^**	n.a.	N=2 (0.9%)			

Mean values are given for age, education, income, IQ and age of onset [95% confidence interval]

a Education (Verhage): range 0 (no education), 3-5 (school diploma) to 8 (university degree).

b Gross income a month: 1= minimal or below, 2= above minimal, below modal, 3= above modal

c Other diagnosis: depression, bipolar disorder, autism, adjustment disorder, anorexia nervosa, personality disorder and obsessive compulsive disorder

### IQ

**Figure 1:**
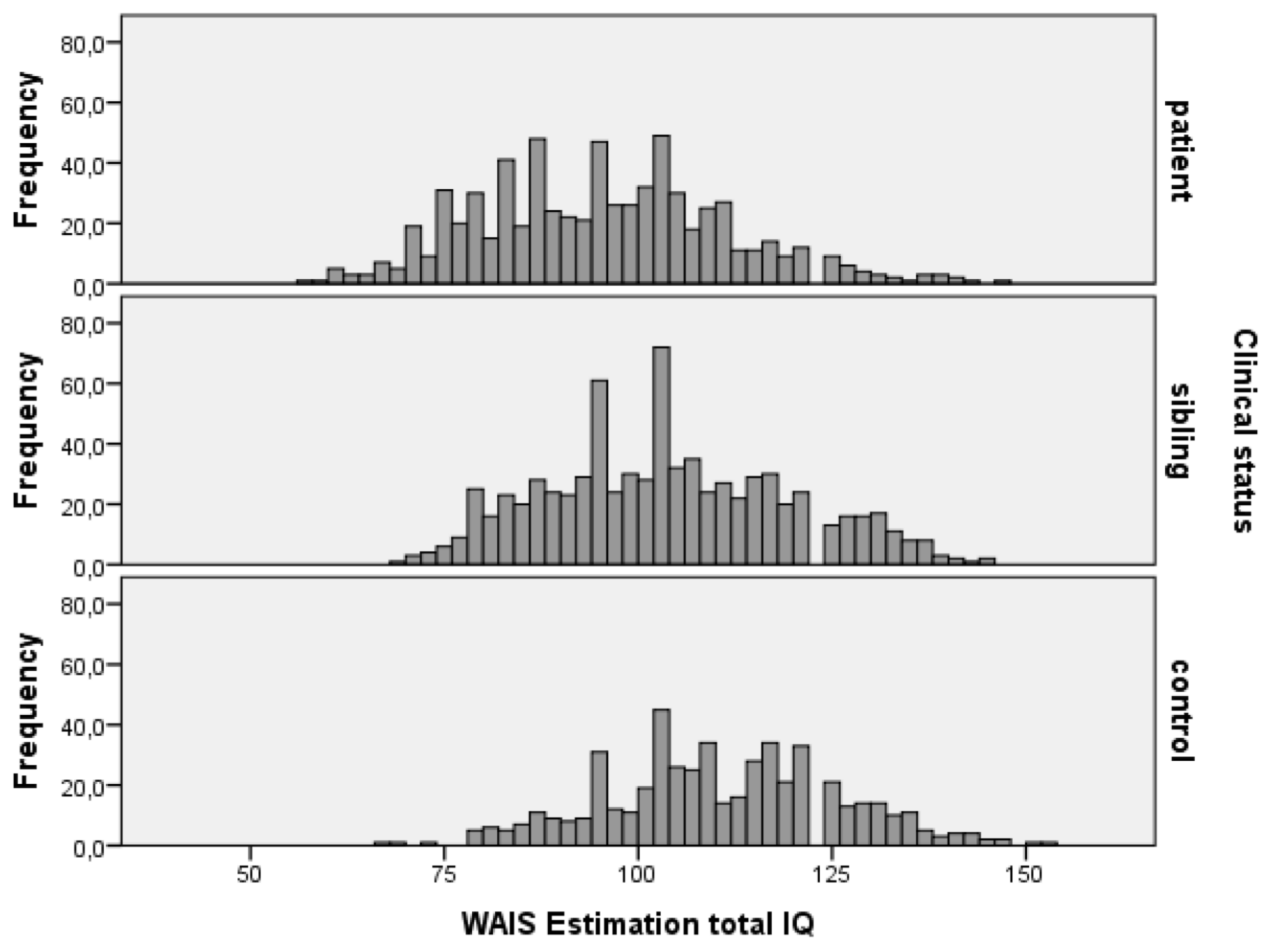
IQ distribution for patients, siblings and controls. Mean IQ patients=94.69 [93.49-95.88], mean IQ siblings=103.06 [101.95-104.17], mean IQ controls=110.17 [108.88-111.46].

**Figure 2:**
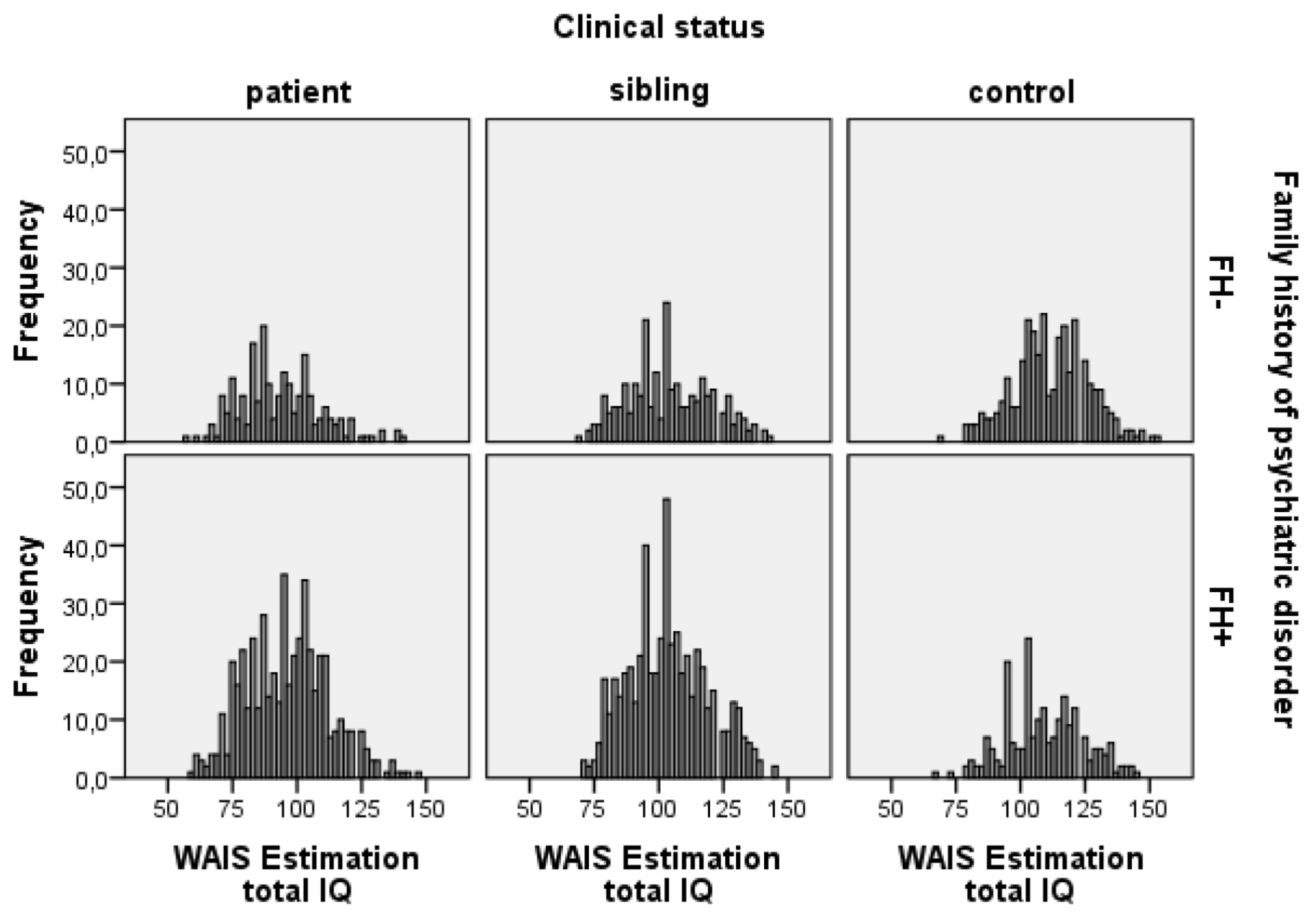
IQ distribution for patients, siblings and controls with and without affected relatives. FH+ patients mean IQ=95.52 [94.08-96.95], FH- patients mean IQ=92.72 [90.57-94.88]; FH+ siblings mean IQ=102.98 [101.65-104.30], FH- siblings mean IQ=103.24 [101.19-105.29]; FH+ controls mean IQ=110.17 [108.71-110.76], FH- controls mean IQ=111.19 [109.52-112.85].

**Figure 3:**
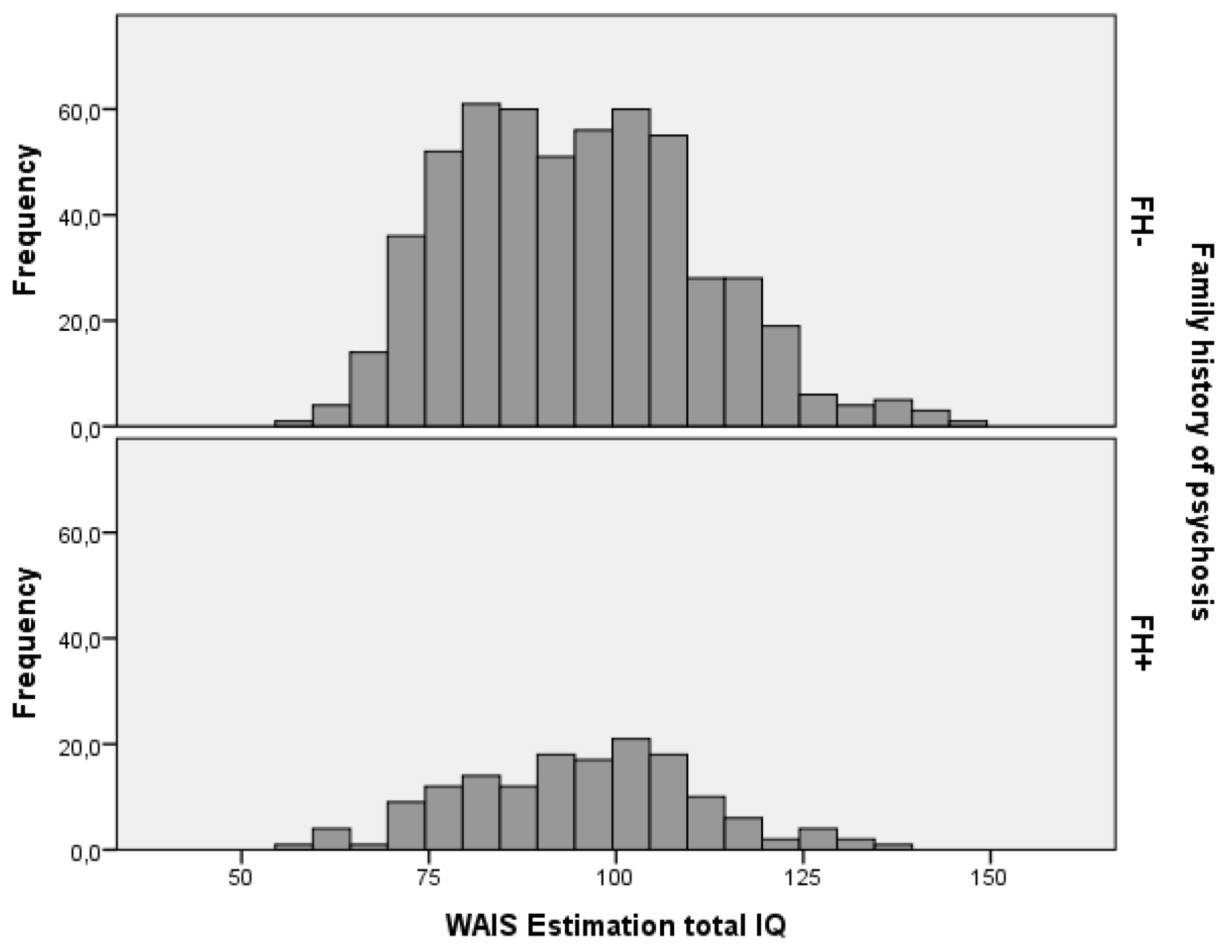
IQ distribution for patients with and without relatives with psychosis. FH- patients (n=544) mean IQ=94.53 [93.17-95.89], FH+ patients (n=152) mean IQ=95.23 [92.71-97.75].

**Figure 4:**
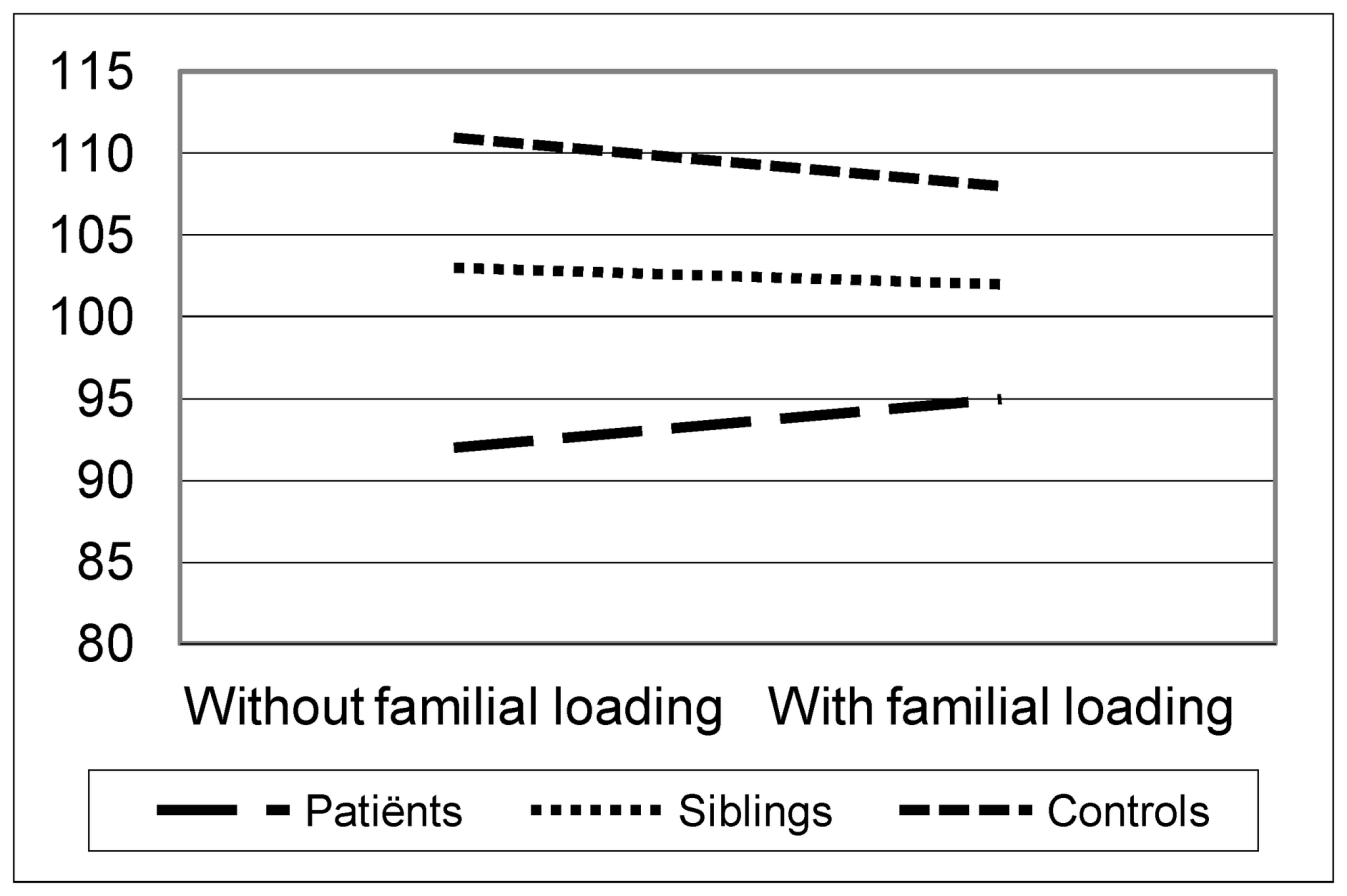
Interaction between family history and subject status Schizophrenia patients obtained lower IQ scores (mean=94.69 [93.49-95.88]) compared to controls (mean=110.17 [108.88-111.46]) with intermediate scores in the siblings of schizophrenia patients (mean=103.06 [101.95-104.17]). A significant interaction was found between family history of psychiatric disorder and clinical status (F(2,1086.87)=4.17; p=.016). Patients with a positive family history of psychiatric disorder obtained higher intelligence scores compared to patients with no family history (mean IQ scores are 95.52 [94.08-96.95] and 92.72 [90.57-94.88], respectively) with an opposite effect in controls (mean IQ scores are 108.71 [106.67-110.76] and 111.19 [109.52-112.85], respectively). No significant difference was found between siblings of schizophrenia patients with or without a positive family history (mean IQ scores are 102.98 [101.65-104.30] and 103.24 [101.19-105.29], respectively).

Schizophrenia patients obtained lower IQ scores (mean=94.69 [93.53-95.85]) compared to controls (mean=110.17 [108.82-111.51]) with intermediate scores in the siblings of schizophrenia patients (mean=103.06 [101.95-104.16], F(2,1978)=149.2; p<.001). IQ distribution of patients, siblings and controls is shown in figures 1, 2 and 3. Comparison of IQ scores in individuals with and without a family history of psychiatric disorder revealed a significant interaction between family history and clinical status (F(2,1086.87)= 4.17; p=.016). In patients, the presence of psychiatric disorder in the pedigree was associated with higher IQ scores, while in controls the presence of psychiatric disorder was associated with lower IQ scores (see Figure 4). No significant difference was found between siblings of schizophrenia patients with or without a positive family history.

The analysis was repeated in the subset of control families where the mothers provided family history information. The results revealed similar IQ differences in controls with and without family history for psychiatric disorder with highest IQ scores in controls without a family history for psychiatric disorder.

Post hoc analyses were conducted to investigate if the significant differences in total IQ scores can be explained by specific subscales. These analyses demonstrate that the mean differences between familial loading groups had the same direction for all subscales and are therefore the result of differences in cognitive performance in all domains (data not shown).

## Discussion

The relationship between family history of psychiatric disorder and IQ was analyzed in 712 schizophrenia proband families (696 patients and 766 siblings) and 427 healthy control families (517 subjects). Patients obtained lower IQ scores compared to controls with intermediate scores in the siblings. A significant interaction was found between family history of psychiatric disorder and clinical status in the sense that patients with a positive family history of psychiatric disorder obtained higher IQ scores, while controls with a positive family history of psychiatric disorder obtained lower IQ scores. No significant association between family history and IQ was found in the siblings of patients with psychosis.

Patients with one or more affected relatives obtained higher IQ scores compared to patients who are the only affected subject in the family. This finding is inconsistent with previous studies that found no difference in full scale IQ between patients with or without affected relatives [19,20] or found higher IQ scores in patients without affected relatives in the family [18]. These inconsistencies could be explained by differences in recruitment strategies, sample size, definition of full scale IQ and study design. The most important difference between this study and previous studies is that this study focuses on family history for all psychiatric disorders and not only on psychosis. Limiting the definition of family history to psychosis could have underestimated the psychopathology risk in families. Since impaired cognitive functioning is found in other psychiatric disorders besides psychosis, some of the individuals with impaired cognitive functioning due to their family history for psychiatry in general but not for psychosis would previously have been assigned to an incorrect group. This is strengthened by a sensitivity analysis in which family history was restricted to psychosis only. This analysis demonstrated no significant differences between IQ scores for patients and siblings with and without family history of psychosis, consistent with previous research [12,19,20]. Unfortunately, controls could not be included in this analysis since controls were selected only if no psychosis was present in their first and second degree relatives.

The lower IQ scores in patients with a negative family history of psychiatric disorder may suggest that the etiology in these patients involves environmental or genetic factors which are unique to this patient and which are not present in the other individuals in the same pedigree. Possible factors include severe environmental stressors including brain injury or premature birth which have been shown to be associated with the risk of schizophrenia [37,38] as well as impaired intellectual functioning [39,40]. Alternatively, genetic factors such as de novo Copy Number Variants (CNVs) may be involved as CNV burden has been found to be increased in schizophrenia patients [41] and is also associated with impaired intellectual functioning. We may hypothesize that patients with no family history of psychiatric disorder more often carry a de novo CNVs compared to patients with a family history of psychiatric disorder. This is supported by the findings of Xu and colleagues who report significantly increased rates of de novo CNVs in schizophrenia patients who are the only affected subject in the family compared to schizophrenia patients with multiple affected relatives [42]. Since increased CNV rates have also been found to be associated with intellectual disability [43], it could be hypothesized that the lower IQ scores in patients without affected relatives reflect an increased vulnerability to CNVs in this group. The notion that individual risk factors together cause the onset of psychosis is supported by recent articles stating that schizophrenia can be seen as a neurodevelopmental disorder in which brain development is disturbed by (epi)genetic and environmental factors eventually leading to psychosis [21,44]. This neurodevelopmental approach is applicable for a variety of psychiatric disorders. The group of patients without relatives affected by any psychiatric disorder included in this study forms an interesting sample to learn about differences between psychosis and other related psychiatric disorders. Studying a sample like this is useful in unraveling the clinical, etiological and pathological overlap from schizophrenia with other psychiatric disorders [45].

There was no association between family history of psychiatric disorder and IQ in siblings. This finding is consistent with a previous study that demonstrated no differences in IQ scores between first degree relatives with one or more relatives with schizophrenia [12]. IQ scores in siblings appear to be influenced by the genetic vulnerability for the disorder since siblings - as a group - consistently obtain lower IQ scores as compared to healthy controls. Since the association between IQ scores and family history of psychiatric disorder in siblings is not extensively investigated, more research is needed to further address this question.

Several limitations of this study should be considered. First, the operationalization of family history of psychiatric disorder is limited to psychosis, depression, mania and substance abuse. Although these are common disorders, family history scores could have been different when anxiety and developmental disorders such as autism would have been taken into account. Second, it was not documented which family member provided family history information. In patients, this would usually be the mother of the patient. Informant differences could potentially influence the reliability of the collected information since previous research indicates that a person with a central position in the pedigree should be the informant of choice [46]. However, the impact on the findings of this study is expected to be small because we would not expect this informant bias to be different in families with and without a family history of psychiatric disorder. This was also supported by the similar results that were found in the subset of control families where the mothers provided family history information. Third, family history information was based on an informant and not on clinical interviews. This might have caused some bias which is strengthened by the fact that 8% of the siblings and 10% of the controls in the group without family history had a psychiatric diagnosis as assessed by the CASH interview. It should be noticed that it is possible to meet DSM IV criteria for a psychiatric diagnosis according to the CASH interview without the experience of being mentally ill. This is confirmed by the fact that the majority of the siblings (90%) and half of the controls (52%) with a psychiatric diagnosis didn’t receive treatment. Finally, the assessment of IQ was based on the short form of the WAIS. Although this short form seems to be a valid instrument and short forms has been used in previous studies, the estimation of IQ based on the full WAIS might have been slightly different since admission of this short form somewhat overestimates IQ scores [35].

This study provides evidence for increased cognitive impairment in schizophrenia patients without a family history of psychiatric disorder compared to patients with a positive family history. Impaired IQ scores are found in different psychiatric disorders and a variety of psychiatric disorders are often present in relatives of patients with schizophrenia. Following the example of Mortensen et al [23], the definition of family history was broadened by focusing on psychiatric disorder in general irrespective of a specific diagnosis. In controls the presence of psychiatric disorder was associated with lower IQ scores, while in patients the presence of psychiatric disorder in the pedigree was associated with higher IQ scores. We hypothesize that the lower IQ in patients with no family history of psychiatric disorder are explained by environmental or genetic factors which are unique in a pedigree. Possible factors include severe environmental stressors including premature birth or brain injury and genetic factors (e.g. Copy Number Variants). These factors may increase the risk for schizophrenia but are also associated with intellectual disability. Further research is needed to test this hypothesis.
